# 2-[(*E*)-4-Diethyl­amino-2-hy­droxy­benzyl­idene]hydrazinecarboxamide

**DOI:** 10.1107/S1600536812000311

**Published:** 2012-01-14

**Authors:** Hoong-Kun Fun, Chin Wei Ooi, Shridhar Malladi, Arun M. Isloor, Kammasandra N. Shivananda

**Affiliations:** a X-ray Crystallography Unit, School of Physics, Universiti Sains Malaysia, 11800 USM, Penang, Malaysia; b Medicinal Chemistry Division, Department of Chemistry, National Institute of Technology-Karnataka, Surathkal, Mangalore 575 025, India; c Schulich Faculty of Chemistry, Technion Israel Institute of Technology, Haifa-Israel

## Abstract

Two mol­ecules make up the asymmetric unit of the title compound, C_12_H_18_N_4_O_2_, and both feature an intra­molecular O—H⋯N hydrogen bond, which generates an *S*(6) ring. The diethyl­amino group of one of the mol­ecules is disordered over two sets of sites in a 0.59 (2):0.41 (2) ratio. In the crystal, N—H⋯O hydrogen bonds link the mol­ecules into sheets lying parallel to the *ac* plane and C—H⋯π inter­actions are also observed.

## Related literature

For a related structure and background references to semicarbazides and semicarbazones, see: Fun *et al.* (2011 ▶). For hydrogen-bond motifs, see: Bernstein *et al.* (1995 ▶). For reference bond-length data, see: Allen *et al.* (1987 ▶).
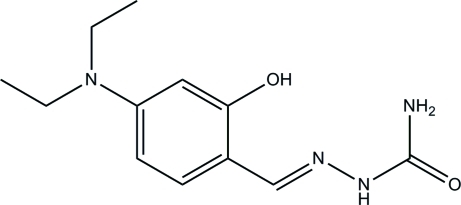


## Experimental

### 

#### Crystal data

C_12_H_18_N_4_O_2_
*M* *_r_* = 250.30
Triclinic, 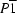

*a* = 8.794 (2) Å
*b* = 12.532 (3) Å
*c* = 14.292 (5) Å
α = 112.911 (7)°
β = 96.033 (7)°
γ = 107.296 (5)°
*V* = 1340.8 (7) Å^3^
*Z* = 4
Mo *K*α radiation
μ = 0.09 mm^−1^
*T* = 296 K
0.35 × 0.12 × 0.03 mm

#### Data collection

Bruker APEX DUO CCD diffractometer
Absorption correction: multi-scan (*SADABS*; Bruker, 2009 ▶) *T*_min_ = 0.970, *T*_max_ = 0.998
15373 measured reflections
4567 independent reflections
2179 reflections with *I* > 2σ(*I*)
*R*_int_ = 0.082

#### Refinement

*R*[*F* ^2^ > 2σ(*F* ^2^)] = 0.068
*wR*(*F* ^2^) = 0.220
*S* = 1.00
4567 reflections
378 parameters
H-atom parameters constrained
Δρ_max_ = 0.37 e Å^−3^
Δρ_min_ = −0.23 e Å^−3^

### 

Data collection: *APEX2* (Bruker, 2009 ▶); cell refinement: *SAINT* (Bruker, 2009 ▶); data reduction: *SAINT*; program(s) used to solve structure: *SHELXTL* (Sheldrick, 2008 ▶); program(s) used to refine structure: *SHELXTL*; molecular graphics: *SHELXTL*; software used to prepare material for publication: *SHELXTL* and *PLATON* (Spek, 2009 ▶).

## Supplementary Material

Crystal structure: contains datablock(s) global, I. DOI: 10.1107/S1600536812000311/hb6571sup1.cif

Structure factors: contains datablock(s) I. DOI: 10.1107/S1600536812000311/hb6571Isup2.hkl

Supplementary material file. DOI: 10.1107/S1600536812000311/hb6571Isup3.cml

Additional supplementary materials:  crystallographic information; 3D view; checkCIF report

## Figures and Tables

**Table 1:** Hydrogen-bond geometry (Å, °) *Cg*1 is the centroid of the C1*B*–C6*B* benzene ring.

*D*—H⋯*A*	*D*—H	H⋯*A*	*D*⋯*A*	*D*—H⋯*A*
O1*A*—H1*O*1⋯N2*A*	0.87	1.79	2.608 (4)	157
O1*B*—H2*O*1⋯N2*B*	0.87	1.91	2.654 (5)	142
N3*A*—H1*N*3⋯O2*A*^i^	0.95	1.90	2.832 (4)	168
N3*B*—H2*N*3⋯O2*B*^ii^	0.99	1.87	2.837 (4)	168
N4*A*—H1*N*4⋯O1*B*	0.79	2.40	3.077 (5)	144
N4*A*—H2*N*4⋯O2*B*^iii^	0.90	2.01	2.901 (5)	172
N4*B*—H3*N*4⋯O2*A*^iv^	0.78	2.14	2.911 (5)	167
N4*B*—H4*N*4⋯O1*A*	0.89	2.20	2.962 (5)	144
C9*A*—H9*AB*⋯*Cg*1^v^	0.97	2.83	3.733 (19)	156
C10*X*—H10*F*⋯*Cg*1^v^	0.96	2.71	3.46 (3)	136

Symmetry codes: (i) 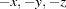; (ii) 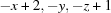; (iii) 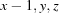; (iv) 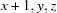; (v) 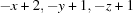.

**Fig. 1.**
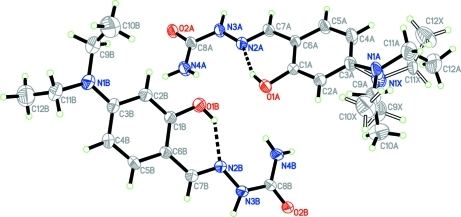
The molecular structure of the title compound, showing two crystallographically independent molecules with 30% probability displacement ellipsoids. Intramolecular hydrogen bonds and minor component of disorder are shown as dashed line and open bonds, respectively.

**Fig. 2.**
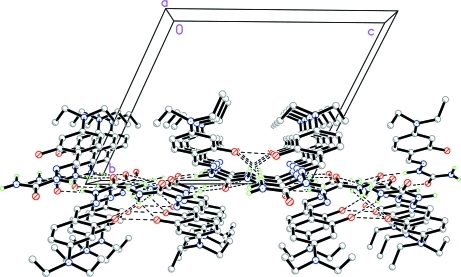
The crystal packing of the title compound, showing a two-dimensional network parallel to the ac plane. Intermolecular hydrogen bonds are shown as dashed lines.

## supplementary crystallographic information

### Comment

As part of our ongoing studies of semicarbazides (Fun *et al.*, 2011), we now describe the structure of the title compound, (I).

The asymmetric unit of (I) consists of two crystallographically independent molecules A and B as shown in Fig. 1. The diethylamino group (N1/C9–C12) in the molecule A is observed to be disordered over two positions with a site-occupancy ratio of 0.59 (2): 0.41 (2). The intramolecular O1A—H1O1···N2A and O1B—H2O1···N2B hydrogen bonds generate S(6) ring motifs (Bernstein *et al.*, 1995) in both molecules. The bond lengths (Allen *et al.*, 1987) and angles are within normal ranges and are comparable to a related structure (Fun *et al.*, 2011).

In the crystal (Fig. 2), N3A—H1N3···O2A, N3B—H2N3···O2B, N4B—H3N4···O2A, N4A—H2N4···O2B, N4A—H1N4···O1B and N4B—H4N4···O1A hydrogen bonds (Table 1) link the molecules into two-dimensional network parallel to the *ac* plane. The crystal structure is further consolidated by C—H···π interactions, involving the centroid of the benzene ring (C1B–C6B; *Cg*1; Table 1).

### Experimental

Semicarbazide hydrochloride (0.86 g, 7.70 mmol) and freshly recrystallized sodium acetate (0.77 g, 9.40 mmol) were dissolved in water (10 ml). The reaction mixture was stirred at room temperature for 10 minutes. To this, *N*,*N*-diethylaminosalicylaldehyde (1.396 g, 7.23 mmol) was added and the mixture was shaken well. A little alcohol was added to dissolve the turbidity. The mixture was shaken for a further 10 minutes and allowed to stand. The title compound crystallizes out on standing for 6 h. The separated crystals were filtered, washed with cold water and recrystallized from ethanol. Yield: 1.4 g, 77.43%. *M*.p. 508–510 K.

### Refinement

All N and O bound H atoms were located from the difference map and were fixed at their found positions with *U*_iso_(H) = 1.2 *U*_eq_(N) and 1.5 *U*_eq_(O). [N–H = 0.7896–0.9855 Å and O–H = 0.8662 and 0.8740 Å]. The hydrogen atoms bounded to C atoms were positioned geometrically [C–H = 0.93, 0.96, and 0.97 Å] with *U*_iso_(H) = 1.2 or 1.5 *U*_eq_(C). A rotating group model was applied to the methyl groups. The diethylamino group in one molecule was modelled as disordered over two sets of sites in a 0.59 (2): 0.41 (2) ratio.

### Crystal data

**Table:**

C_12_H_18_N_4_O_2_	*Z* = 4
*M**_r_* = 250.30	*F*(000) = 536
Triclinic, *P*1	*D*_x_ = 1.240 Mg m^−^^3^
Hall symbol: -P 1	Mo *K*α radiation, λ = 0.71073 Å
*a* = 8.794 (2) Å	Cell parameters from 1473 reflections
*b* = 12.532 (3) Å	θ = 2.5–21.7°
*c* = 14.292 (5) Å	µ = 0.09 mm^−^^1^
α = 112.911 (7)°	*T* = 296 K
β = 96.033 (7)°	Plate, colourless
γ = 107.296 (5)°	0.35 × 0.12 × 0.03 mm
*V* = 1340.8 (7) Å^3^	

### Data collection

**Table:**

Bruker APEX DUO CCD diffractometer	4567 independent reflections
Radiation source: fine-focus sealed tube	2179 reflections with *I* > 2σ(*I*)
graphite	*R*_int_ = 0.082
φ and ω scans	θ_max_ = 25.0°, θ_min_ = 1.9°
Absorption correction: multi-scan (*SADABS*; Bruker, 2009)	*h* = −10→10
*T*_min_ = 0.970, *T*_max_ = 0.998	*k* = −14→14
15373 measured reflections	*l* = −16→16

### Refinement

**Table:**

Refinement on *F*^2^	Secondary atom site location: difference Fourier map
Least-squares matrix: full	Hydrogen site location: inferred from neighbouring sites
*R*[*F*^2^ > 2σ(*F*^2^)] = 0.068	H-atom parameters constrained
*wR*(*F*^2^) = 0.220	*w* = 1/[σ^2^(*F*_o_^2^) + (0.1117*P*)^2^] where *P* = (*F*_o_^2^ + 2*F*_c_^2^)/3
*S* = 1.00	(Δ/σ)_max_ < 0.001
4567 reflections	Δρ_max_ = 0.37 e Å^−^^3^
378 parameters	Δρ_min_ = −0.23 e Å^−^^3^
0 restraints	Extinction correction: *SHELXTL* (Sheldrick, 2008), Fc^*^=kFc[1+0.001xFc^2^λ^3^/sin(2θ)]^-1/4^
Primary atom site location: structure-invariant direct methods	Extinction coefficient: 0.020 (5)

### Special details

**Table:**

Geometry. All e.s.d.'s (except the e.s.d. in the dihedral angle between two l.s. planes) are estimated using the full covariance matrix. The cell e.s.d.'s are taken into account individually in the estimation of e.s.d.'s in distances, angles and torsion angles; correlations between e.s.d.'s in cell parameters are only used when they are defined by crystal symmetry. An approximate (isotropic) treatment of cell e.s.d.'s is used for estimating e.s.d.'s involving l.s. planes.
Refinement. Refinement of *F*^2^ against ALL reflections. The weighted *R*-factor *wR* and goodness of fit *S* are based on *F*^2^, conventional *R*-factors *R* are based on *F*, with *F* set to zero for negative *F*^2^. The threshold expression of *F*^2^ > σ(*F*^2^) is used only for calculating *R*-factors(gt) *etc*. and is not relevant to the choice of reflections for refinement. *R*-factors based on *F*^2^ are statistically about twice as large as those based on *F*, and *R*- factors based on ALL data will be even larger.

### Fractional atomic coordinates and isotropic or equivalent isotropic displacement parameters (Å^2^)

**Table:**

	*x*	*y*	*z*	*U*_iso_*/*U*_eq_	Occ. (<1)
O1A	0.6655 (3)	0.1779 (3)	0.2307 (2)	0.0701 (8)	
H1O1	0.5606	0.1487	0.2032	0.105*	
O2A	−0.0328 (3)	−0.0381 (2)	0.10768 (18)	0.0570 (7)	
N1A	1.139 (2)	0.3932 (17)	0.1487 (15)	0.071 (4)	0.59 (2)
C9A	1.2352 (15)	0.4443 (17)	0.2568 (12)	0.069 (4)	0.59 (2)
H9AA	1.1634	0.4606	0.3032	0.082*	0.59 (2)
H9AB	1.3202	0.5237	0.2735	0.082*	0.59 (2)
C10A	1.3145 (18)	0.3656 (12)	0.2799 (14)	0.091 (4)	0.59 (2)
H10A	1.3591	0.4007	0.3542	0.136*	0.59 (2)
H10B	1.4014	0.3617	0.2448	0.136*	0.59 (2)
H10C	1.2345	0.2830	0.2556	0.136*	0.59 (2)
C11A	1.2166 (18)	0.4446 (19)	0.0825 (13)	0.089 (5)	0.59 (2)
H11A	1.3022	0.5253	0.1271	0.107*	0.59 (2)
H11B	1.1350	0.4577	0.0415	0.107*	0.59 (2)
C12A	1.290 (2)	0.3647 (14)	0.0100 (13)	0.112 (5)	0.59 (2)
H12A	1.3312	0.4013	−0.0344	0.169*	0.59 (2)
H12B	1.2070	0.2833	−0.0325	0.169*	0.59 (2)
H12C	1.3780	0.3579	0.0499	0.169*	0.59 (2)
N1X	1.137 (4)	0.338 (2)	0.126 (3)	0.082 (7)	0.41 (2)
C9X	1.248 (2)	0.355 (2)	0.2198 (17)	0.082 (6)	0.41 (2)
H9XA	1.3568	0.3644	0.2079	0.098*	0.41 (2)
H9XB	1.2082	0.2828	0.2328	0.098*	0.41 (2)
C10X	1.258 (3)	0.469 (2)	0.313 (2)	0.102 (7)	0.41 (2)
H10D	1.3259	0.4764	0.3746	0.153*	0.41 (2)
H10E	1.1499	0.4616	0.3230	0.153*	0.41 (2)
H10F	1.3055	0.5414	0.3026	0.153*	0.41 (2)
C11X	1.230 (2)	0.372 (2)	0.0587 (18)	0.079 (6)	0.41 (2)
H11C	1.1754	0.3083	−0.0126	0.095*	0.41 (2)
H11D	1.3367	0.3662	0.0756	0.095*	0.41 (2)
C12X	1.262 (3)	0.496 (2)	0.0576 (17)	0.110 (7)	0.41 (2)
H12D	1.3144	0.4983	0.0024	0.166*	0.41 (2)
H12E	1.3331	0.5608	0.1238	0.166*	0.41 (2)
H12F	1.1600	0.5070	0.0460	0.166*	0.41 (2)
N2A	0.3762 (3)	0.1003 (3)	0.1066 (2)	0.0506 (8)	
N3A	0.2069 (3)	0.0468 (3)	0.0751 (2)	0.0549 (8)	
H1N3	0.1478	0.0326	0.0095	0.066*	
N4A	0.2015 (4)	0.0214 (3)	0.2272 (3)	0.0679 (10)	
H1N4	0.2962	0.0631	0.2488	0.081*	
H2N4	0.1436	−0.0001	0.2695	0.081*	
C1A	0.7295 (4)	0.2133 (3)	0.1602 (3)	0.0518 (10)	
C2A	0.8972 (4)	0.2705 (4)	0.1830 (3)	0.0627 (11)	
H2AA	0.9617	0.2838	0.2453	0.075*	
C3A	0.9731 (5)	0.3092 (4)	0.1144 (3)	0.0661 (12)	
C4A	0.8712 (5)	0.2833 (4)	0.0200 (3)	0.0695 (12)	
H4AA	0.9174	0.3053	−0.0286	0.083*	
C5A	0.7041 (5)	0.2256 (4)	−0.0015 (3)	0.0612 (11)	
H5AA	0.6401	0.2099	−0.0650	0.073*	
C6A	0.6255 (4)	0.1896 (3)	0.0660 (3)	0.0456 (9)	
C7A	0.4491 (4)	0.1316 (3)	0.0425 (3)	0.0512 (9)	
H7AA	0.3859	0.1162	−0.0210	0.061*	
C8A	0.1193 (5)	0.0088 (3)	0.1376 (3)	0.0509 (9)	
O1B	0.5150 (3)	0.1937 (3)	0.4058 (2)	0.0696 (8)	
H2O1	0.6021	0.1737	0.4043	0.104*	
O2B	1.0082 (3)	−0.0267 (2)	0.36887 (19)	0.0561 (7)	
N1B	0.2108 (5)	0.3598 (4)	0.6416 (3)	0.0821 (12)	
N2B	0.7297 (3)	0.1094 (3)	0.4683 (2)	0.0492 (8)	
N3B	0.8512 (4)	0.0593 (3)	0.4610 (2)	0.0528 (8)	
H2N3	0.8853	0.0467	0.5226	0.063*	
N4B	0.8368 (4)	0.0353 (3)	0.2920 (3)	0.0699 (10)	
H3N4	0.8593	0.0056	0.2389	0.084*	
H4N4	0.7739	0.0806	0.3003	0.084*	
C1B	0.4775 (4)	0.2152 (3)	0.4999 (3)	0.0496 (9)	
C2B	0.3629 (5)	0.2704 (4)	0.5218 (3)	0.0606 (11)	
H2BA	0.3142	0.2893	0.4723	0.073*	
C3B	0.3186 (5)	0.2987 (4)	0.6184 (3)	0.0578 (10)	
C4B	0.3893 (5)	0.2639 (4)	0.6874 (3)	0.0580 (11)	
H4BA	0.3599	0.2783	0.7506	0.070*	
C5B	0.5022 (5)	0.2086 (3)	0.6638 (3)	0.0525 (10)	
H5BA	0.5465	0.1861	0.7120	0.063*	
C6B	0.5538 (4)	0.1842 (3)	0.5718 (3)	0.0446 (9)	
C7B	0.6773 (4)	0.1298 (3)	0.5521 (3)	0.0495 (9)	
H7BA	0.7206	0.1086	0.6016	0.059*	
C8B	0.9039 (5)	0.0206 (4)	0.3727 (3)	0.0517 (9)	
C9B	0.1244 (7)	0.3852 (6)	0.5621 (5)	0.0962 (17)	
H9BA	0.1035	0.3187	0.4926	0.115*	
H9BB	0.0194	0.3879	0.5756	0.115*	
C10B	0.2252 (8)	0.5042 (6)	0.5667 (5)	0.122 (2)	
H10G	0.1741	0.5160	0.5106	0.183*	
H10H	0.3325	0.5040	0.5598	0.183*	
H10I	0.2352	0.5709	0.6328	0.183*	
C11B	0.1968 (6)	0.4216 (5)	0.7525 (4)	0.0918 (16)	
H11E	0.2921	0.4322	0.8009	0.110*	
H11F	0.1945	0.5034	0.7675	0.110*	
C12B	0.0490 (7)	0.3475 (5)	0.7673 (5)	0.1114 (19)	
H12G	0.0302	0.3956	0.8329	0.167*	
H12H	0.0618	0.2742	0.7679	0.167*	
H12I	−0.0430	0.3234	0.7111	0.167*	

### Atomic displacement parameters (Å^2^)

**Table:**

	*U*^11^	*U*^22^	*U*^33^	*U*^12^	*U*^13^	*U*^23^
O1A	0.0498 (16)	0.119 (2)	0.0724 (19)	0.0315 (15)	0.0236 (14)	0.0708 (18)
O2A	0.0424 (15)	0.0861 (19)	0.0444 (15)	0.0184 (13)	0.0128 (12)	0.0342 (14)
N1A	0.043 (5)	0.092 (10)	0.066 (7)	−0.007 (7)	0.010 (4)	0.048 (8)
C9A	0.051 (6)	0.079 (9)	0.055 (8)	0.006 (5)	0.003 (6)	0.026 (8)
C10A	0.096 (8)	0.096 (8)	0.080 (9)	0.049 (6)	0.010 (7)	0.031 (7)
C11A	0.062 (7)	0.101 (13)	0.099 (10)	0.017 (8)	0.020 (6)	0.050 (10)
C12A	0.099 (9)	0.158 (12)	0.109 (10)	0.059 (8)	0.049 (7)	0.074 (9)
N1X	0.061 (9)	0.095 (16)	0.098 (16)	0.012 (13)	0.022 (10)	0.062 (15)
C9X	0.052 (10)	0.095 (12)	0.078 (14)	0.009 (8)	0.019 (9)	0.031 (11)
C10X	0.100 (13)	0.088 (13)	0.086 (15)	0.003 (10)	0.009 (12)	0.033 (14)
C11X	0.056 (9)	0.117 (16)	0.087 (14)	0.027 (10)	0.041 (8)	0.065 (13)
C12X	0.115 (14)	0.092 (15)	0.096 (13)	−0.002 (11)	0.008 (10)	0.050 (10)
N2A	0.0417 (17)	0.063 (2)	0.053 (2)	0.0194 (14)	0.0159 (15)	0.0309 (16)
N3A	0.0400 (17)	0.081 (2)	0.0468 (19)	0.0167 (15)	0.0135 (14)	0.0355 (17)
N4A	0.0446 (18)	0.109 (3)	0.057 (2)	0.0194 (18)	0.0134 (16)	0.050 (2)
C1A	0.049 (2)	0.069 (3)	0.058 (3)	0.0273 (19)	0.0218 (19)	0.042 (2)
C2A	0.044 (2)	0.094 (3)	0.064 (3)	0.022 (2)	0.014 (2)	0.051 (2)
C3A	0.044 (2)	0.095 (3)	0.068 (3)	0.017 (2)	0.019 (2)	0.050 (3)
C4A	0.059 (3)	0.091 (3)	0.062 (3)	0.017 (2)	0.025 (2)	0.042 (2)
C5A	0.054 (2)	0.084 (3)	0.047 (2)	0.016 (2)	0.0153 (19)	0.037 (2)
C6A	0.041 (2)	0.060 (2)	0.043 (2)	0.0175 (17)	0.0136 (16)	0.0292 (18)
C7A	0.053 (2)	0.066 (2)	0.040 (2)	0.0213 (19)	0.0141 (18)	0.0277 (19)
C8A	0.051 (2)	0.064 (2)	0.039 (2)	0.0183 (19)	0.0141 (18)	0.0258 (18)
O1B	0.0695 (18)	0.114 (2)	0.0463 (17)	0.0531 (17)	0.0241 (14)	0.0401 (16)
O2B	0.0627 (16)	0.0853 (19)	0.0542 (16)	0.0473 (15)	0.0307 (13)	0.0453 (14)
N1B	0.095 (3)	0.126 (3)	0.074 (3)	0.080 (3)	0.047 (2)	0.057 (2)
N2B	0.0505 (18)	0.065 (2)	0.0472 (19)	0.0304 (16)	0.0174 (15)	0.0310 (16)
N3B	0.0597 (19)	0.077 (2)	0.0467 (19)	0.0416 (17)	0.0217 (15)	0.0378 (16)
N4B	0.085 (2)	0.121 (3)	0.050 (2)	0.070 (2)	0.0355 (18)	0.055 (2)
C1B	0.047 (2)	0.067 (2)	0.037 (2)	0.0248 (19)	0.0114 (17)	0.0225 (19)
C2B	0.060 (2)	0.085 (3)	0.051 (2)	0.038 (2)	0.017 (2)	0.036 (2)
C3B	0.057 (2)	0.069 (3)	0.058 (3)	0.034 (2)	0.025 (2)	0.028 (2)
C4B	0.059 (2)	0.073 (3)	0.055 (3)	0.031 (2)	0.027 (2)	0.032 (2)
C5B	0.060 (2)	0.065 (2)	0.042 (2)	0.023 (2)	0.0192 (18)	0.0322 (19)
C6B	0.044 (2)	0.050 (2)	0.039 (2)	0.0169 (17)	0.0096 (16)	0.0198 (17)
C7B	0.049 (2)	0.058 (2)	0.046 (2)	0.0223 (18)	0.0116 (18)	0.0255 (19)
C8B	0.057 (2)	0.069 (3)	0.046 (2)	0.030 (2)	0.0189 (19)	0.036 (2)
C9B	0.100 (4)	0.128 (5)	0.113 (5)	0.075 (4)	0.060 (4)	0.073 (4)
C10B	0.141 (5)	0.137 (5)	0.110 (5)	0.067 (5)	0.046 (4)	0.059 (4)
C11B	0.090 (4)	0.121 (4)	0.114 (4)	0.070 (3)	0.062 (3)	0.069 (4)
C12B	0.110 (5)	0.130 (5)	0.100 (4)	0.049 (4)	0.041 (4)	0.049 (4)

### Geometric parameters (Å, °)

**Table:**

O1A—C1A	1.360 (4)	C3A—C4A	1.400 (6)
O1A—H1O1	0.8662	C4A—C5A	1.371 (5)
O2A—C8A	1.239 (4)	C4A—H4AA	0.9300
N1A—C3A	1.418 (19)	C5A—C6A	1.381 (5)
N1A—C11A	1.46 (3)	C5A—H5AA	0.9300
N1A—C9A	1.46 (2)	C6A—C7A	1.442 (5)
C9A—C10A	1.48 (3)	C7A—H7AA	0.9300
C9A—H9AA	0.9700	O1B—C1B	1.361 (4)
C9A—H9AB	0.9700	O1B—H2O1	0.8740
C10A—H10A	0.9600	O2B—C8B	1.227 (4)
C10A—H10B	0.9600	N1B—C3B	1.376 (5)
C10A—H10C	0.9600	N1B—C9B	1.487 (6)
C11A—C12A	1.49 (2)	N1B—C11B	1.506 (6)
C11A—H11A	0.9700	N2B—C7B	1.288 (4)
C11A—H11B	0.9700	N2B—N3B	1.385 (4)
C12A—H12A	0.9600	N3B—C8B	1.350 (5)
C12A—H12B	0.9600	N3B—H2N3	0.9855
C12A—H12C	0.9600	N4B—C8B	1.340 (4)
N1X—C3A	1.35 (3)	N4B—H3N4	0.7808
N1X—C11X	1.44 (4)	N4B—H4N4	0.8898
N1X—C9X	1.47 (4)	C1B—C2B	1.381 (5)
C9X—C10X	1.50 (4)	C1B—C6B	1.406 (5)
C9X—H9XA	0.9700	C2B—C3B	1.414 (5)
C9X—H9XB	0.9700	C2B—H2BA	0.9300
C10X—H10D	0.9600	C3B—C4B	1.385 (5)
C10X—H10E	0.9600	C4B—C5B	1.371 (5)
C10X—H10F	0.9600	C4B—H4BA	0.9300
C11X—C12X	1.50 (3)	C5B—C6B	1.387 (5)
C11X—H11C	0.9700	C5B—H5BA	0.9300
C11X—H11D	0.9700	C6B—C7B	1.442 (5)
C12X—H12D	0.9600	C7B—H7BA	0.9300
C12X—H12E	0.9600	C9B—C10B	1.464 (7)
C12X—H12F	0.9600	C9B—H9BA	0.9700
N2A—C7A	1.287 (4)	C9B—H9BB	0.9700
N2A—N3A	1.378 (4)	C10B—H10G	0.9600
N3A—C8A	1.369 (4)	C10B—H10H	0.9600
N3A—H1N3	0.9445	C10B—H10I	0.9600
N4A—C8A	1.330 (5)	C11B—C12B	1.446 (7)
N4A—H1N4	0.7896	C11B—H11E	0.9700
N4A—H2N4	0.8989	C11B—H11F	0.9700
C1A—C2A	1.373 (5)	C12B—H12G	0.9600
C1A—C6A	1.415 (5)	C12B—H12H	0.9600
C2A—C3A	1.399 (5)	C12B—H12I	0.9600
C2A—H2AA	0.9300		
			
C1A—O1A—H1O1	102.4	C5A—C6A—C7A	122.3 (3)
C3A—N1A—C11A	121.8 (15)	C1A—C6A—C7A	122.0 (3)
C3A—N1A—C9A	121.6 (15)	N2A—C7A—C6A	122.1 (3)
C11A—N1A—C9A	116.2 (14)	N2A—C7A—H7AA	118.9
N1A—C9A—C10A	116 (2)	C6A—C7A—H7AA	118.9
N1A—C9A—H9AA	108.3	O2A—C8A—N4A	122.5 (3)
C10A—C9A—H9AA	108.3	O2A—C8A—N3A	119.0 (3)
N1A—C9A—H9AB	108.3	N4A—C8A—N3A	118.5 (3)
C10A—C9A—H9AB	108.3	C1B—O1B—H2O1	110.1
H9AA—C9A—H9AB	107.4	C3B—N1B—C9B	121.2 (4)
N1A—C11A—C12A	114 (2)	C3B—N1B—C11B	121.1 (4)
N1A—C11A—H11A	108.7	C9B—N1B—C11B	117.0 (4)
C12A—C11A—H11A	108.7	C7B—N2B—N3B	116.0 (3)
N1A—C11A—H11B	108.7	C8B—N3B—N2B	122.0 (3)
C12A—C11A—H11B	108.7	C8B—N3B—H2N3	124.0
H11A—C11A—H11B	107.6	N2B—N3B—H2N3	113.6
C3A—N1X—C11X	126 (3)	C8B—N4B—H3N4	117.2
C3A—N1X—C9X	124 (2)	C8B—N4B—H4N4	120.9
C11X—N1X—C9X	110 (2)	H3N4—N4B—H4N4	121.8
N1X—C9X—C10X	110 (3)	O1B—C1B—C2B	116.7 (3)
N1X—C9X—H9XA	109.6	O1B—C1B—C6B	121.5 (3)
C10X—C9X—H9XA	109.6	C2B—C1B—C6B	121.8 (3)
N1X—C9X—H9XB	109.6	C1B—C2B—C3B	120.8 (4)
C10X—C9X—H9XB	109.6	C1B—C2B—H2BA	119.6
H9XA—C9X—H9XB	108.1	C3B—C2B—H2BA	119.6
C9X—C10X—H10D	109.5	N1B—C3B—C4B	122.4 (4)
C9X—C10X—H10E	109.5	N1B—C3B—C2B	120.3 (4)
H10D—C10X—H10E	109.5	C4B—C3B—C2B	117.3 (4)
C9X—C10X—H10F	109.5	C5B—C4B—C3B	120.8 (4)
H10D—C10X—H10F	109.5	C5B—C4B—H4BA	119.6
H10E—C10X—H10F	109.5	C3B—C4B—H4BA	119.6
N1X—C11X—C12X	121 (3)	C4B—C5B—C6B	123.5 (4)
N1X—C11X—H11C	107.1	C4B—C5B—H5BA	118.3
C12X—C11X—H11C	107.1	C6B—C5B—H5BA	118.3
N1X—C11X—H11D	107.1	C5B—C6B—C1B	115.8 (3)
C12X—C11X—H11D	107.1	C5B—C6B—C7B	121.4 (3)
H11C—C11X—H11D	106.8	C1B—C6B—C7B	122.8 (3)
C11X—C12X—H12D	109.5	N2B—C7B—C6B	122.3 (3)
C11X—C12X—H12E	109.5	N2B—C7B—H7BA	118.9
H12D—C12X—H12E	109.5	C6B—C7B—H7BA	118.9
C11X—C12X—H12F	109.5	O2B—C8B—N4B	123.1 (4)
H12D—C12X—H12F	109.5	O2B—C8B—N3B	119.6 (3)
H12E—C12X—H12F	109.5	N4B—C8B—N3B	117.4 (3)
C7A—N2A—N3A	116.3 (3)	C10B—C9B—N1B	109.9 (5)
C8A—N3A—N2A	120.3 (3)	C10B—C9B—H9BA	109.7
C8A—N3A—H1N3	117.9	N1B—C9B—H9BA	109.7
N2A—N3A—H1N3	121.8	C10B—C9B—H9BB	109.7
C8A—N4A—H1N4	119.2	N1B—C9B—H9BB	109.7
C8A—N4A—H2N4	118.2	H9BA—C9B—H9BB	108.2
H1N4—N4A—H2N4	120.5	C9B—C10B—H10G	109.5
O1A—C1A—C2A	117.6 (3)	C9B—C10B—H10H	109.5
O1A—C1A—C6A	120.6 (3)	H10G—C10B—H10H	109.5
C2A—C1A—C6A	121.8 (3)	C9B—C10B—H10I	109.5
C1A—C2A—C3A	121.3 (4)	H10G—C10B—H10I	109.5
C1A—C2A—H2AA	119.3	H10H—C10B—H10I	109.5
C3A—C2A—H2AA	119.3	C12B—C11B—N1B	110.8 (5)
N1X—C3A—C2A	119.4 (14)	C12B—C11B—H11E	109.5
N1X—C3A—C4A	121.0 (15)	N1B—C11B—H11E	109.5
C2A—C3A—C4A	117.1 (4)	C12B—C11B—H11F	109.5
C2A—C3A—N1A	120.6 (9)	N1B—C11B—H11F	109.5
C4A—C3A—N1A	121.0 (9)	H11E—C11B—H11F	108.1
C5A—C4A—C3A	120.6 (4)	C11B—C12B—H12G	109.5
C5A—C4A—H4AA	119.7	C11B—C12B—H12H	109.5
C3A—C4A—H4AA	119.7	H12G—C12B—H12H	109.5
C4A—C5A—C6A	123.5 (4)	C11B—C12B—H12I	109.5
C4A—C5A—H5AA	118.3	H12G—C12B—H12I	109.5
C6A—C5A—H5AA	118.3	H12H—C12B—H12I	109.5
C5A—C6A—C1A	115.7 (3)		
			
C3A—N1A—C9A—C10A	84.1 (19)	O1A—C1A—C6A—C7A	2.3 (6)
C11A—N1A—C9A—C10A	−102.9 (19)	C2A—C1A—C6A—C7A	−178.9 (3)
C3A—N1A—C11A—C12A	−86.4 (19)	N3A—N2A—C7A—C6A	179.3 (3)
C9A—N1A—C11A—C12A	101 (2)	C5A—C6A—C7A—N2A	−178.6 (4)
C3A—N1X—C9X—C10X	−67 (3)	C1A—C6A—C7A—N2A	1.3 (6)
C11X—N1X—C9X—C10X	106 (2)	N2A—N3A—C8A—O2A	179.5 (3)
C3A—N1X—C11X—C12X	71 (3)	N2A—N3A—C8A—N4A	−2.0 (5)
C9X—N1X—C11X—C12X	−101 (3)	C7B—N2B—N3B—C8B	172.5 (3)
C7A—N2A—N3A—C8A	179.0 (3)	O1B—C1B—C2B—C3B	−178.7 (3)
O1A—C1A—C2A—C3A	179.6 (4)	C6B—C1B—C2B—C3B	0.4 (6)
C6A—C1A—C2A—C3A	0.7 (6)	C9B—N1B—C3B—C4B	−174.0 (4)
C11X—N1X—C3A—C2A	176 (2)	C11B—N1B—C3B—C4B	16.0 (7)
C9X—N1X—C3A—C2A	−13 (3)	C9B—N1B—C3B—C2B	6.8 (7)
C11X—N1X—C3A—C4A	15 (3)	C11B—N1B—C3B—C2B	−163.2 (4)
C9X—N1X—C3A—C4A	−174.7 (18)	C1B—C2B—C3B—N1B	176.5 (4)
C11X—N1X—C3A—N1A	−84 (5)	C1B—C2B—C3B—C4B	−2.7 (6)
C9X—N1X—C3A—N1A	87 (5)	N1B—C3B—C4B—C5B	−176.8 (4)
C1A—C2A—C3A—N1X	−164.5 (14)	C2B—C3B—C4B—C5B	2.4 (6)
C1A—C2A—C3A—C4A	−2.2 (7)	C3B—C4B—C5B—C6B	0.3 (6)
C1A—C2A—C3A—N1A	164.9 (10)	C4B—C5B—C6B—C1B	−2.6 (5)
C11A—N1A—C3A—N1X	91 (5)	C4B—C5B—C6B—C7B	177.8 (3)
C9A—N1A—C3A—N1X	−97 (5)	O1B—C1B—C6B—C5B	−178.7 (3)
C11A—N1A—C3A—C2A	−174.0 (15)	C2B—C1B—C6B—C5B	2.3 (5)
C9A—N1A—C3A—C2A	−1(2)	O1B—C1B—C6B—C7B	0.8 (5)
C11A—N1A—C3A—C4A	−7(2)	C2B—C1B—C6B—C7B	−178.2 (3)
C9A—N1A—C3A—C4A	165.2 (13)	N3B—N2B—C7B—C6B	178.5 (3)
N1X—C3A—C4A—C5A	163.9 (14)	C5B—C6B—C7B—N2B	−178.2 (3)
C2A—C3A—C4A—C5A	1.9 (7)	C1B—C6B—C7B—N2B	2.2 (5)
N1A—C3A—C4A—C5A	−165.2 (10)	N2B—N3B—C8B—O2B	−177.7 (3)
C3A—C4A—C5A—C6A	−0.1 (7)	N2B—N3B—C8B—N4B	1.7 (5)
C4A—C5A—C6A—C1A	−1.4 (6)	C3B—N1B—C9B—C10B	−88.2 (6)
C4A—C5A—C6A—C7A	178.6 (4)	C11B—N1B—C9B—C10B	82.1 (5)
O1A—C1A—C6A—C5A	−177.8 (3)	C3B—N1B—C11B—C12B	−103.9 (5)
C2A—C1A—C6A—C5A	1.1 (6)	C9B—N1B—C11B—C12B	85.7 (6)

### Hydrogen-bond geometry (Å, °)

**Table:**

Cg1 is the centroid of the C1*B*–C6*B* benzene ring.

**Table:**

*D*—H···*A*	*D*—H	H···*A*	*D*···*A*	*D*—H···*A*
O1A—H1O1···N2A	0.87	1.79	2.608 (4)	157
O1B—H2O1···N2B	0.87	1.91	2.654 (5)	142
N3A—H1N3···O2A^i^	0.95	1.90	2.832 (4)	168
N3B—H2N3···O2B^ii^	0.99	1.87	2.837 (4)	168
N4A—H1N4···O1B	0.79	2.40	3.077 (5)	144
N4A—H2N4···O2B^iii^	0.90	2.01	2.901 (5)	172
N4B—H3N4···O2A^iv^	0.78	2.14	2.911 (5)	167
N4B—H4N4···O1A	0.89	2.20	2.962 (5)	144
C9A—H9AB···Cg1^v^	0.97	2.83	3.733 (19)	156
C10X—H10F···Cg1^v^	0.96	2.71	3.46 (3)	136

Symmetry codes: (i) −*x*, −*y*, −*z*; (ii) −*x*+2, −*y*, −*z*+1; (iii) *x*−1, *y*, *z*; (iv) *x*+1, *y*, *z*; (v) −*x*+2, −*y*+1, −*z*+1.
